# Overexpression of heparan sulfate 6-*O*-sulfotransferase-2 in colorectal cancer

**DOI:** 10.3892/mco.2013.151

**Published:** 2013-07-23

**Authors:** SHIGERU HATABE, HIDEHARU KIMURA, TOKUZO ARAO, HIROAKI KATO, HIDETOSHI HAYASHI, TOMOYUKI NAGAI, KAZUKO MATSUMOTO, MARCO DE VELASCO, YOSHIHIKO FUJITA, GO YAMANOUCHI, MASAO FUKUSHIMA, YASUHIDE YAMADA, AKIHIKO ITO, KIYOTAKA OKUNO, KAZUTO NISHIO

**Affiliations:** 1 Department of Surgery, Kinki University School of Medicine, Osaka-Sayama, Osaka 589-8511, Japan; 2 Department of Genome Biology, Kinki University School of Medicine, Osaka-Sayama, Osaka 589-8511, Japan; 3 Sumitomo Bakelite Co., Ltd., Nishi-ku, Kobe 651-2241, Japan; 4 Department of Medical Oncology, National Cancer Center Hospital, Chuo-ku, Tokyo 104-0045, Japan; 5 Department of Pathology, Kinki University School of Medicine, Osaka-Sayama, Osaka 589-8511, Japan

**Keywords:** heparan sulfate 6-*O*-sulfotransferase-2, heparan sulfate, colorectal cancer

## Abstract

The heparan sulfate sulfotransferase gene family catalyzes the transfer of sulfate groups to heparan sulfate and regulates various growth factor-receptor signaling pathways. However, the involvement of this gene family in cancer biology has not been elucidated. It was demonstrated that the heparan sulfate D-glucosaminyl 6-*O*-sulfotransferase-2 (*HS6ST2*) gene is overexpressed in colorectal cancer (CRC) and its clinical significance in patients with CRC was investigated. The mRNA levels of *HS6ST2* in clinical CRC samples and various cancer cell lines were assessed using a microarray analysis and quantitative RT-PCR, respectively. An immunohistochemical (IHC) analysis of the HS6ST2 protein was performed using 102 surgical specimens of CRC. The correlations between the HS6ST2 expression status and clinicopathological characteristics were then evaluated. *HS6ST2* mRNA was significantly overexpressed by 37-fold in CRC samples compared to paired colonic mucosa. High levels of *HS6ST2* mRNA expression were also observed in colorectal, esophageal and lung cancer cell lines. The IHC analysis demonstrated that HS6ST2 was expressed in the cytoplasmic region of CRC cells, but not in normal colonic mucosal cells. Positive staining for HS6ST2 was detected in 40 patients (39.2%). There was no significant association between the clinicopathological characteristics and HS6ST2 expression. However, positive staining for HS6ST2 was associated with a poor survival (P=0.074, log-rank test). In conclusion, HS6ST2 was found to be overexpressed in CRC and its expression tended to be a poor prognostic factor, although the correlation was not significant. These findings indicate that *HS6ST2* may be a novel cancer-related marker that may provide insight into the glycobiology of CRC.

## Introduction

Colorectal cancer (CRC) is one of the most common types of cancer worldwide. The incidence of CRC is estimated to be 1,000,000 new cases annually and the incidence has continuously increased over the last 25 years (1). Although some progress has been made, CRC remains a major cause of mortality and further insight into the biology of CRC is required to improve CRC patient outcome. In order to develop an optimal strategy for selecting CRC patients that would benefit from adjuvant chemotherapy following surgery, reliable markers for predicting relapse have been extensively investigated. Therefore, there is a need for the identification of molecular characteristics of CRC cells that may be used to develop specific biomarkers for tumor growth and prognosis.

In a previous study, we identified novel biomarkers by investigating overexpressed genes in CRC cells compared to paired normal colonic mucosa, using a microarray analysis (2). In the course of that analysis, the heparan sulfate D-glucosaminyl 6-*O*-sulfotransferase-2 (*HS6ST2*) gene was identified as a candidate biomarker for CRC.

*HS6ST2* is a member of the *HS6ST2* gene family, which catalyzes the transfer of sulfate groups from adenosine 3′-phosphate, 5′-phosphosulphate to the C-6 (exocyclic carbon) of the glucosamine residue in heparan sulfate proteoglycans (HSPGs). HSPGs are known to be involved in the progression of malignant tumors (3,4). The overexpression of HSPGs was previously correlated with a worse stage of breast cancer (5). Cell surface HSPGs, particularly syndecan-1, are overexpressed in the majority of pancreatic cancer tissues and surrounding metastatic lesions (6). The role of HSPGs in cancer cells may be to increase growth factor signaling. The 6-*O*-sulfation of heparan sulfate (HS) promotes the formation of the trimolecular complex comprising a growth factor, its receptor and HS. The most thoroughly investigated growth factor is fibroblast growth factor (FGF) (7–10). The hepatocyte growth factor and the vascular endothelial growth factor (VEGF) are other growth factors known to form trimolecular complexes. These growth factors play a critical role in cancer development via the promotion of cell growth and angiogenesis. A previous study demonstrated that *HS6ST2* gene expression is regulated by the transforming growth factor-β (TGF-β) and the Wnt signaling pathways in normal murine mammary gland epithelial cells (11). In ovarian cancer, HS6ST1 and HS6ST2 were found to be strongly expressed by tumor cells, although only HS6ST1 was detected in endothelial cells (12). As regards the biological function of HS6ST2, activation of HS6ST2 was observed in pancreatic cancer cells and the gene silencing of endogenous HS6ST2 expression inhibited cell growth, invasion, migration and tumorigenicity (13). HS6ST2 was also investigated as an important gene for TGF-β-induced IL-11 production in highly metastatic MDA-MB-231 (SA) cancer cells (14). Thus, emerging evidence suggests an association between HS6ST2 expression and the biological function of cancer cells. However, HS6ST2 expression and its clinical significance have not been elucidated.

It was hypothesized that HS6ST2 plays an important role in the progression of CRC and that HS6ST2 expression may be a useful biomarker for the prognosis of CRC patients. In the present study, the mRNA and protein expression of HS6ST2 was evaluated in surgical CRC specimens.

## Materials and methods

### Quantitative reverse transcription-polymerase chain reaction (qRT-PCR)

*HS6ST2* mRNA expression in cancer cell lines was measured using qRT-PCR. The total RNA extracted from cultured cells was converted to cDNA using SuperScript™ III Reverse Transcriptase (Life Technologies, Carlsbad, CA, USA). qPCR was performed using SYBR^®^ Premix Ex Taq™ (Takara Bio, Inc., Shiga, Japan) at a final volume of 25 μl, starting with a 3-min template denaturation step at 95ºC, followed by 40 cycles of 15 sec at 95ºC and 1 min at 60ºC. The primers were designed by Takara Bio, Inc. and the sequences were as follows: HS6ST2, forward: 5′-CTCCTGTCTCTGTCTTAT-3′ and reverse: 5′-GCAATAGATTTATTAAGTATCCC-3′. To normalize the possible variations in sample concentration, glyceraldehyde-3-phosphate dehydrogenase (*GAPDH*) was used as a housekeeping control. The *HS6ST2/GAPDH* mRNA ratio was calculated for each cell line to evaluate the relative mRNA expression.

### Human tissue samples

This retrospective study was approved by the Institutional Review Board of Kinki University. BN961 (Biomax, Rockville, MD, USA), which is a multiple normal tissue microarray with 24 normal human organs, including normal colonic tissue, was used for the normal tissues. Archived, formalin-fixed, paraffin-embedded tissues were retrieved from surgically resected (with curative intent) CRC specimens containing the tumor and adjacent normal colonic tissues at Kinki University Hospital. The tissues were cut into 4-μm sections and used for immunohistochemical staining. A total of 102 CRC samples were evaluated and the corresponding patient records, including age at diagnosis, gender, histological findings, tumor location, TNM grade, treatment after surgery, date of surgery and date of death, were collected.

### Immunohistochemical (IHC) analysis

IHC analyses were conducted using the HS6ST2 specific anti-mouse monoclonal antibody, which recognized the epitope corresponding to amino acids 379–459 within the human HS6ST2. The antibody for HS6ST2 was provided by Sumitomo Bakelite Co., Ltd. (Kobe, Japan). Following deparaffinization and rehydration, the sections were treated in 0.01 M citrate buffer (pH 6.0) for 15 min in a pressure cooker. Endogenous peroxidase activity was blocked for 10 min in 3% hydrogen peroxide in methanol. Non-specific binding was blocked by treatment with a blocking reagent for 30 min at room temperature. Sections were incubated with anti-HS6ST2 antibody as a primary antibody overnight at 4ºC and then incubated with a biotinylated secondary antibody for 1 h at room temperature, followed by incubation with the peroxidase-conjugated biotin-avidin complex (Vectastain^®^ ABC kit; Vector Laboratories, Burlingame, CA, USA) for 30 min. Peroxidase activity was then visualized by adding 3,3′-diaminobenzidine substrate solution and the reaction was stopped by washing with water. The sections were then counterstained with hematoxylin. Scores were obtained based on the proportion of cells with a positive signal (0–100%). The IHC grades were defined as: negative, no cells with a positive signal; mild, <25% of cells with a positive signal; strong, ≥25% of cells with a positive signal.

### Microarray analysis

Gene expression data for paired CRC and non-cancerous colonic mucosa samples were obtained using a microarray analysis as previously described (2).

### Statistical analysis

The HS6ST2 expression status was used to divide the samples into the HS6ST2-positive and -negative groups, and clinical factors, such as age (<65 vs. ≥65 years), gender, tumor location (colon vs. rectum), TNM stage (0, I and II vs. III and IV), with T grade (Tis 1 and 2 vs. 3 and 4), N grade (0 vs. 1, 2 and 3) and M grade (0 vs. 1) being compared between the groups, using the Mann-Whitney U test. The survival time analyses (from the time of surgery until death) were performed using patients who had received adjuvant chemotherapy after surgery. Univariate analyses were used to evaluate the clinical factors. The survival distributions were estimated using the Kaplan-Meier method and the differences between the two groups were compared using the log-rank test. P<0.05 was considered to indicate a statistically significant difference. The data analyses were performed using IBM^®^ SPSS^®^ Statistics 19 software (IBM Corporation, Somers, NY, USA).

## Results

### Overexpression of HS6ST2 mRNA in CRC

**Figure 1:**
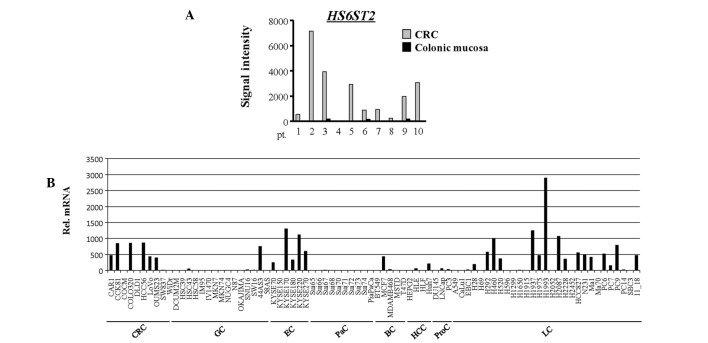
mRNA expression levels of heparan sulfate 6-*O*-sulfotransferase 2 (*HS6ST2*) in 10 paired colorectal cancer (CRC) and non-cancerous colonic mucosal samples and in various cancer cell lines. (A) Signal intensities of *HS6ST2* were obtained from a microarray analysis. *HS6ST2* was overexpressed in CRC (gray bars) compared to the paired mucosal samples (black bars) in almost all the patients. (B) The mRNA expression levels of *HS6ST2* were assessed using quantitative reverse transcription-polymerase chain reaction in a panel of 83 cancer cell lines. Pt, patient number; Rel. mRNA, normalized mRNA expression levels (*HS6ST2/GAPD* × 10^6^); GC, gastric cancer; EC, esophageal cancer; PaC, pancreatic cancer; BC, breast cancer; HCC, hepatocellular carcinoma; ProC, prostate cancer; LC, lung cancer.

*HS6ST2* mRNA expression levels were evaluated in 10 paired CRC and non-cancerous colonic mucosal samples, using previous microarray analysis data (2). The *HS6ST2* mRNA was overexpressed by 37-fold in the CRC compared to the paired colonic mucosa samples (P=0.01, Fig. 1A). These results clearly demonstrated the overexpression of *HS6ST2* mRNA in CRC and suggested that HS6ST2 expression is upregulated during the development of CRC.

### HS6ST2 mRNA expression in cancer cell lines

To gain insight into the expression profile of *HS6ST2* mRNA in cancer cells, a panel of 83 cancer cell lines from various organ sites was investigated [10, CRC; 15, gastric cancer (GC); 6, esophageal cancer (EC); 10, pancreatic cancer (PaC); 5, breast cancer (BC); 4, hepatocellular carcinoma (HCC); 3, prostate cancer (ProC); and 30, lung cancer (LC)], using qRT-PCR. A high expression level of *HS6ST2* mRNA was observed in 6 of the 10 CRC cell lines, 5 of the 6 EC cell lines and 17 of the 30 LC cell lines. Almost none of the PaC and GC cell lines expressed *HS6ST2* (Fig. 1B).

### HS6ST2 expression in normal organ tissues

**Figure 2:**
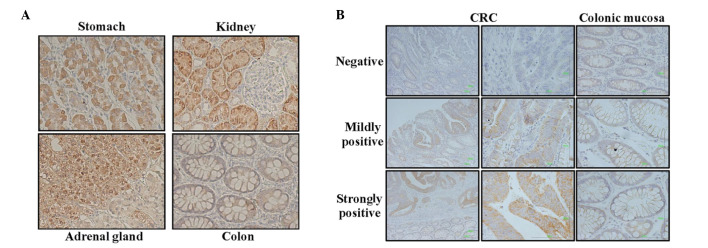
Immunohistochemical (IHC) analysis for heparan sulfate 6-*O*-sulfotransferase-2 (HS6ST2) in (A) normal tissues and (B) representative images of colorectal cancer (CRC) tissues. IHC stainings of the stomach, adrenal gland and kidney are shown, all of which are representative examples of a strong expression. No expression was observed in normal colonic tissues. The results of the normal tissue array are summarized in Table I. Representative IHC staining in colonic tissues of CRC patients is defined as negative, mildly positive and strongly positive, according to the expression level and staining intensity.

To investigate the protein expression of HS6ST2, the HS6ST2 expression levels were evaluated in 24 normal organ tissues using IHC analysis. HS6ST2 expression was detected in 9 of the 24 organs (stomach, liver, adrenal gland, bronchus, breast, ovary, uterus, kidney and skin; Table I). A strong positive expression was observed in the normal tissues of the stomach, adrenal gland and kidney, whereas no expression was observed in normal colonic tissue (Fig. 2A).

### HS6ST2 expression in CRC

The HS6ST2 expression levels in 102 surgical specimens of CRC were then investigated, using IHC analysis. HS6ST2 staining was observed in the CRC cells and not in the normal colonic mucosal cells. The expression of HS6ST2 was localized in the cytoplasm in positive cases, but not in the nucleus or the cell membrane. Representative microphotographs of the expression are shown in Fig. 2B. HS6ST2 expression was detected in 40 (39.2%) of the 102 cases, of which 24 cases (23.5%) were classified as mildly positive and 16 (15.7%) as strongly positive. The results indicate that HS6ST2 expression varied widely from negative to strongly positive in the clinical CRC samples.

The correlation between HS6ST2 expression and clinical characteristics, such as age, gender, tumor location, degree of differentiation, lymph vessel invasion, venous invasion and TNM grade was also evaluated (Table II). No significant differences were observed between the groups with a positive and negative HS6ST2 expression.

### Overall survival (OS) analysis according to HS6ST2 expression status

To evaluate the clinical effect of HS6ST2 expression on postoperative OS, a survival analysis was performed for 50 patients who had received adjuvant 5FU-based chemotherapy. Lymph vessel invasion, TNM grade and distant metastasis (M grade) were significantly associated with OS (P=0.012, 0.006 and 0.001, respectively) (Table III). Of note, HS6ST2 expression tended to be a poor prognostic factor, although the association was not statistically significant (P=0.084).

**Figure 3:**
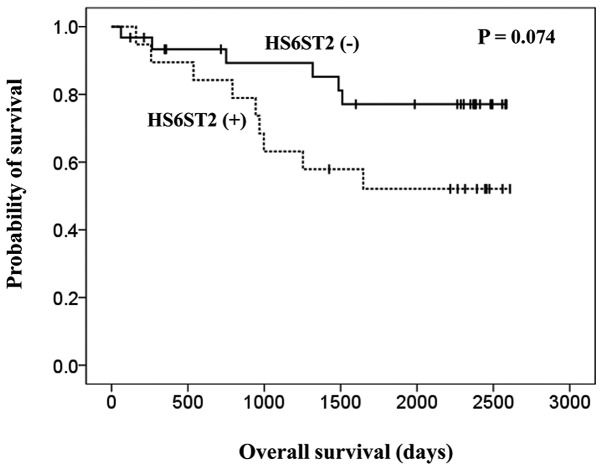
Kaplan-Meier curves for overall survival. Patients were divided into two groups according to their heparan sulfate 6-*O*-sulfotransferase-2 (HS6ST2) expression status (dotted line, HS6ST2-positive group; straight line, HS6ST2-negative group). The P-value was calculated using the log-rank test.

The Kaplan-Meier estimates for OS with regard to the HS6ST2 expression status revealed a shorter OS for the patients with a positive HS6ST2 expression (P=0.074, log-rank test, Fig. 3). The survival analysis demonstrated that the prognosis of patients with a positive HS6ST2 expression was distinctly better compared to that of the patients without HS6ST2 expression; however, the difference was not statistically significant. These results indicated that HS6ST2 expression is associated with a poor outcome after surgery among patients receiving adjuvant chemotherapy.

## Discussion

The overexpression of *HS6ST2* mRNA and protein in CRC cells was demonstrated in this study. The sulfation pattern within the S-domains contributes to the structure of heparan sulfate and may create specific binding sites for protein ligands (15). The interaction of heparan sulfate with protein ligands is often essential for the modulation of ligand-receptor binding and may affect the outcome of downstream signaling events (15–17). Previous *in vitro* studies indicated heparan sulfate 6-*O* sulfation as a critical regulatory step in vessel formation (18). Previously, the role of HS6ST in angiogenesis in zebrafish embryos, an excellent model for the study of angiogenesis due to their rapid development, ease of genetic manipulation and comparability to vascular development in mammals, was investigated (18). The activity of HS6ST2 was thus shown to be important for VEGF-mediated angiogenesis. For example, 6-*O* desulfated heparan was previously shown to eliminate the amplifying effect of heparin on the FGF-2 activation of FGFR-1 signaling and to inhibit FGF-2-induced angiogenesis (19). Those findings suggested that the overexpression of HS6ST2 is a promising therapeutic target in CRC. The roles of HS6ST2 expression in angiogenesis and FGFR signaling require further investigations in the clinical setting.

HS6ST2 expression was not significantly associated with the clinical characteristics that were investigated, whereas it tended to be a poor prognostic factor in the survival analysis. These results led us to hypothesize that HS6ST2 expression, as determined by IHC, may be a useful biomarker for CRC diagnosis and prognosis in CRC patients receiving adjuvant chemotherapy. In this study, HS6ST2 expression was detected using an IHC assay with an anti-mouse monoclonal antibody, although the *HS6ST2* mRNA expression levels were analyzed in previous studies (12,13). The IHC assay is a widely used method for visualizing pathological characteristics and confirming a diagnosis. In addition, certain therapeutic decisions on molecular-targeted therapy against solid tumors are based on targeted protein expressions evaluated by IHC. The IHC method used in this study is considered to have a potential as a clinical application.

The IHC analysis revealed that HS6ST2 expression was detected in the cytoplasm of CRC cells in approximately half of the CRC patients. However, it was not expressed by normal colonic mucosal cells. The majority of the enzymes known to be involved in sulfated glycosaminoglycans are Golgi- and rough endoplasmic reticulum-resident proteins that may form multienzyme complexes. Similar to our findings, a previous study by Nagai *et al*(20) demonstrated that HS6ST-1, -2 and -3 colocalized with a Golgi marker under forced expression conditions.

In conclusion, HS6ST2 is overexpressed in CRC and may be associated with a poor survival outcome. Our findings suggest that the HS6ST2 expression status may be a useful biomarker of postoperative outcome in CRC patients.

## Figures and Tables

**Table I:** Immunohistochemical (IHC) staining of heparan sulfate 6-*O*-sulfotransferase-2 (HS6ST2) in various normal tissues.

Organ site	IHC
Cerebrum	−
Cerebellum	−
Heart	−
Esophagus	−
Stomach	++
Small intestine	−
Colon	−
Liver	+
Pancreas	−
Adrenal gland	++
Lung	−
Bronchus	+
Lymph node	−
Spleen	−
Breast	+
Ovary	+
Uterus	+
Kidney	++
Prostate	−
Testis	−
Skeletal muscle	−
Diaphragm	−
Adipose tissue	−
Skin	+

−, negative; +, mildly positive; ++, strongly positive.

**Table II:** Analysis of clinical factors and heparan sulfate 6-*O*-sulfotransferase-2 (HS6ST2) expression.

		HS6ST2 expression		
		
Clinical factors	Total (n=102)	Negative (n=62)	Positive (n=40)	P-value^a^
Age (years)				
Median	63	65	71	0.117
Range	33–90	33–90	33–90	
Gender				
Male	55	29	26	0.103
Female	47	33	14	
Degree of differentiation				
High	53	30	23	0.420
Moderate/poor	49	32	17	
Lymphatic invasion				
Negative	49	30	19	1.000
Positive	53	32	21	
Venous invasion				
Negative	85	49	36	0.181
Positive	17	13	4	
Location				
Colon	57	36	21	0.684
Rectum	45	26	19	
UICC-Stage				
0, I, II	53	31	22	0.687
III, IV	49	31	18	
T grade				
Tis, T1, T2	29	19	10	0.654
T3, T4	73	43	30	
N grade				
N0	56	34	22	1.000
N1, N2	46	28	18	
M grade				
M0	83	52	31	0.445
M1	19	10	9	

a P-values were calculated using the Fisher’s exact probability test, with the exception of age (Mann-Whitney U test).

UICC, Unio Internationalis Contra Cancrum.

**Table III:** Univariate analyses for overall survival.

Variables	Hazard ratio	95% CI	P-value
Age, <65 vs. ≥65	2.254	(0.770–6.598)	0.138
Gender, female vs. male	1.288	(0.467–3.553)	0.625
Location, colon vs. rectum	1.710	(0.584–5.007)	0.328
Differentiation, high vs. moderate/poor	0.973	(0.356–2.715)	0.973
Lymph vessel invasion, negative vs. positive	13.859	(1.781–103.662)	0.012
Venous invasion, negative vs. positive	2.366	(0.804–6.958)	0.118
TNM grade, II vs. III vs. IV	3.277	(1.407–7.634)	0.006
T grade, 1 vs. 2 vs. 3 vs. 4	3.118	(0.909–10.696)	0.071
N grade, 0 vs. 1 vs. 2	2.242	(1.056–4.758)	0.036
M grade, 0 vs. 1	5.757	(2.012–16.474)	0.001
HS6ST2 expression, negative vs. positive	2.487	(0.884–7.003)	0.084

HS6ST2, heparan sulfate 6-*O*-sulfotransferase-2; CI, confidence interval.
